# Functionally Distinct Effects of the C-Terminal Regions of IKKε and TBK1 on Type I IFN Production

**DOI:** 10.1371/journal.pone.0094999

**Published:** 2014-04-10

**Authors:** Yuichiro Nakatsu, Mayumi Matsuoka, Tsung-Hsien Chang, Noriyuki Otsuki, Masahiro Noda, Hirokazu Kimura, Kouji Sakai, Hiroshi Kato, Makoto Takeda, Toru Kubota

**Affiliations:** 1 Department of Virology III, National Institute of Infectious Diseases, Musashi-Murayama, Tokyo, Japan; 2 Department of Bacteriology II, National Institute of Infectious Diseases, Musashi-Murayama, Tokyo, Japan; 3 Department of Medical Education and Research, Kaohsiung Veterans General Hospital, Kaohsiung, Taiwan; 4 Infectious Diseases Surveillance Center, National Institute of Infectious Diseases, Musashi-Murayama, Tokyo, Japan; National Jewish Health and University of Colorado School of Medicine, United States of America

## Abstract

Inhibitor of κB kinase ε (IKKε) and TANK binding kinase 1 (TBK1), so-called non-canonical IKKs or IKK-related kinases, are involved in the cellular innate immunity by inducing type I IFNs. Two kinases commonly phosphorylate transcription factors IRF3 and IRF7 in type I IFN production pathway. In contrast to TBK1, underlying mechanisms of IKKε activation and regions required for activation of downstream molecules are poorly understood. In this study, we investigated regions of IKKε required for the activation of type I IFN promoter specially, by focusing on the C-terminal region. To show the functional significance of the IKKε C-terminal region on type I IFN production, we employed various mutant forms of IKKε and compared to corresponding region of TBK1. We identified the specific regions and residues of IKKε involved in the activation of downstream signaling. Interestingly, corresponding region and residues are not required for activation of downstream signaling by TBK1. The results highlight the importance of the C-terminal region in the functional activity of IKKε in innate immune response and also the difference in activation mechanisms between IKKε and the closely related TBK1.

## Introduction

Viral infection induces the cellular immune responses, which prevent viral propagation and pathogenic activities. A family of inhibitor of κB (IκB) kinase (IKK) is activated in response to pathogen infection, and these kinases control both innate and adaptive immunity [1], [2]. Among them, TANK-binding kinase 1 (TBK1) and IKKε play a key role in the expression of cellular anti-viral effects by inducing type I interferon (IFN) production [3], [4], [5]. Viral nucleic acids are sensed by cellular pattern-recognition receptors (PRRs), such as Toll-like and RIG-I-like receptors as well as cytosolic DNA receptors. These PRRs induce the auto-phosphorylation of Ser 172 located in the T loop of the TBK1 and IKKε kinase domains, which is essential for the enhancement of kinase activity [1], [4], [6], [7], [8], [9]. Activated TBK1 and IKKε phosphorylate IRF3 and IRF7, leading to the nuclear translocation of these transcription factors and subsequent induction of type I IFN promoter activity [3], [4], [10], [11].TBK1 is constitutively expressed in a broad range of cells, while the expression of IKKε is inducible and predominantly takes place in immune cells [12], [13], [14]. Despite these differences, TBK1 and IKKε are found together in a complex and are similarly targeted to the phosphorylation of the C-terminal Ser/Thr rich region of IRF3 and IRF7 [3], [15]. The crystal structure of TBK1 revealed that it has a trimodular architecture with an N-terminal kinase domain (KD), followed by the internally located ubiquitin-like domain (ULD) and the C-terminal helical scaffold dimerization domain (SDD), similar to IKKβ [16], [17], [18]. TBK1 takes on a dimer configuration in crystallized form and dimerization is also required for the activation through auto-phosphorylation of Ser172. The TBK1 dimer is stabilized by an extensive network of interactions among the KD, ULD and SDD, while IKKβ which dimerization is mediated by a C-terminal region of SDD [16], [17], [18].In structural studies on TBK1, a C-terminally truncated fragment was used (residue 1-657), since this TBK1/1-657 fragment forms a dimer both *in vitro* and *in vivo* and is able to induce IRF3 phosphorylation, leading activation of the type I IFN promoter [17], [18]. Therefore, the C-terminal region of TBK1 is dispensable for enhancing TBK1 kinase activity as well as activating downstream signaling, at least in this overexpression system [17], [18], [19], [20]. These observations give rise to the question of whether the activation sequence of IKKε follows the same pattern as TBK1. To investigate this, we focused on the C-terminal region of IKKε and investigated its functional significance upon the dimerization of IKKε, phosphorylation of IRF3 and activation of the IFNβ promoter, respectively.

## Materials and Methods

### Cells Culture

Human embryonic kidney (HEK) 293T and murine L cells were obtained from ATCC (Manassas, VA). 293ET cells were from Invitrogen (Carlsbad, CA). Cells were grown in Dulbecco's modified Eagle's medium (DMEM) supplemented with 10% FCS and antibiotics (Invitrogen).

### Reagents and plasmids

Mouse monoclonal antibodies against FLAG M2 and α-tubulin and anti-FLAG agarose beads were purchased from Sigma (St Louis, MO). Mouse monoclonal antibody for V5-tag and Alexa Fluor 488 and 594-conjugated secondary antibodies were purchased from Invitrogen. Rabbit polyclonal antibody against IRF3 was from Becton Dickinson (Franklin Lakes, NJ). Rabbit antibodies against phospho-IRF3 (pSer396) and phospho-IRF3 (pSer386) were from Cell Signaling Technology (Danvers, MA) and EPITOMICS (Burlingame, CA), respectively. FLAG-human IKKε, FLAG-human TBK1 plasmids were a gift from Dr. Rongtuan Lin (McGill University, Montreal, Canada). To construct mutants for IKKε and TBK1, appropriate substitutions were introduced into the FLAG-human IKKε and FLAG-human TBK1 plasmids using the Quick Change site directed mutagenesis kit (Stratagene, La Jolla, CA). IFNβ promoter-luciferase reporter was a gift from Dr. Takashi Fujita (University of Kyoto, Kyoto, Japan). ISRE-luciferase reporter was from Stratagene.

### Immunoblot analysis

Whole cell extracts were prepared using lysis buffer containing 150 mM NaCl, 50 mM Tris-HCl, pH 7.5, 4 mM EDTA, 0.1% NaDOC, 1% NP40, 0.1% SDS, complete protease inhibitor cocktail (Roche Diagnostics, Tokyo, Japan) and immunoblotted with indicated antibodies.

### Immunoprecipitation

293T cells (3×10^6^) were transfected with a total of 3.3 μg of plasmids DNA using Lipofectamine 2000 (Invitrogen). Twelve h later, cells were lysed using lysis buffer. Lysates were centrifuged and supernatants were incubated with anti-FLAG agarose for overnight with gentle rotation at 4°C. Immune complexes were washed 4 times with lysis buffer. Samples were separated on SDS- PAGE and subjected to immunoblot analysis.

### Luciferase reporter assay

293T cells were plated in 24 well plates at 3×10^4^/0.5 ml and were transiently transfected with IFNβ-luciferase reporter or ISRE-luciferase reporter, and control Renilla luciferase along with indicated plasmids using Lipofectamine 2000 (Invitrogen) according to the manufacturer's recommendations. Eighteen h post transfection, cells were lysed and luciferase activity was measured by using the Dual reporter luciferase assay Kit (Promega) according to the manufacturer's procedure. Renilla luciferase activity was used for normalization.

### Quantitative (q) RT-PCR

Murine L cells (2×10^5^) were transfected with 2.5 μg of indicated plasmids using Lipofectamine 2000. Twenty four h later, total RNA were prepared by using Trizol reagent (Invitrogen) and were reversetranscribed with Transcriptor First Strand cDNA Synthesis Kit (Roche Diagnostic). The amount of IFNβ and hypoxanthine guanine phosphoribosyltransferase (HPRT) cDNA were measured by using Universal ProbeLibrary and LightCycler 480 (Roche Diagnostic) according to the manufacture's instructions. Primers for q-RT-PCR were designed by the ProbeFinder software (Roche Diagnostic).

### Confocal microscopy

293ET cells seeded on coverslips in 12-well plates were transfected with 1 μg of plasmids for FLAG-tagged wt or mutant forms of IKKε (L686S, L697S or K38A) using Lipofectamine 2000 reagent. At 20 h post transfection, cells were fixed and permeabilized with PBS containing 4% paraformaldehyde and 0.5% Triton X-100. Fixed cells were washed with PBS and incubated with antibodies against IRF3 and FLAG for 1 h at room temperature, followed by an Alexa Fluor 488 and 594-conjugated secondary antibodies for 1 h at room temperature. Nuclear DNA was stained with 4′,6′-diamidino-2-phenylindole (DAPI; NacalaiTesque). Immunostained coverslips were mounted onto slides using ProlongGoldAntifade reagent (Invitrogen) and observed using an FV1000D Spectral Type confocal laser-scanning microscope (inverted microscope IX81) (Olympus, Tokyo, Japan).

## Results

### C-terminal region of IKKε is required for IFNβ promoter activity

**Figure 1:**
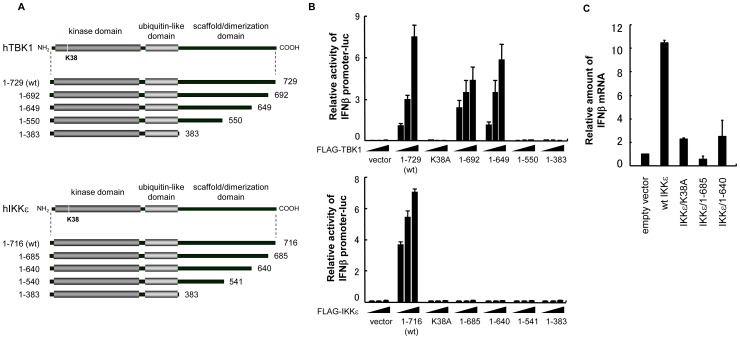
The C-terminal region of IKKε, but not TBK1, is required for IFNβ promoter activity. (A) 293T cells were transfected with an IFNβ promoter-luciferase reporter along with an increasing amount of FLAG-tagged full length IKKε (1-716), TBK1 (1-729), a kinase defective mutant (K38A) or C-terminal deletion mutants, as shown on the left. Cells were lysed 24 h post-transfection and luciferase activities were quantified by normalization with renilla luciferase activity. The values represent the average of three samples +/− SD. (B) L cells were transfected with indicated plasmid and total RNA were prepared at 24 h post-transfection. Relative amount of IFNβ mRNA were quantified by using qRT-PCR by normalization with HPRT mRNA. The values represent the average of three samples +/− SD.

**Figure 2:**
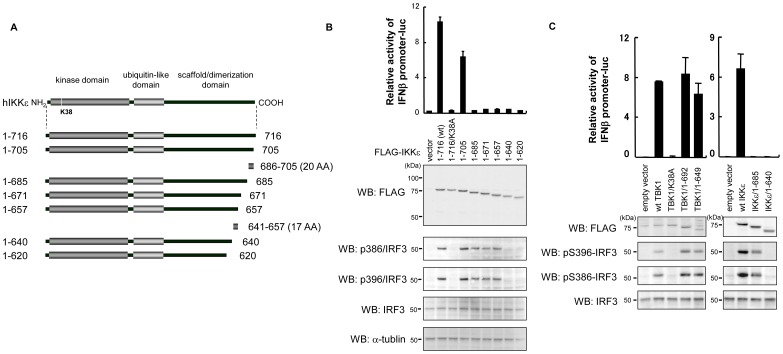
The C-terminal region of IKKε contains two functional domains required for IFNβ promoter activity. (A) Schematic structure of IKKε and C-terminal truncated mutants. The putative functional domains (641–657 and 686–705) are shown. (B) 293T cells were transfected with the IFNβ promoter-luciferase reporter along with the FLAG-tagged full length IKKε (1-716), kinase defective mutant (K38A) or C-terminal deletion mutants presented in (A). Cells were lysed at 24 h post-transfection and luciferase activities were quantified by normalization with renilla luciferase activity. The values represent the average of three samples +/− SD. Cell lysates were also subjected to SDS-PAGE and Western-blotted with the antibodies indicated on the left. (C) 293T cells were transfected with the IFNβ promoter-luciferase reporter along with plasmid for wt or mutant forms of TKB1 and IKKε. Luciferase activities were measured as shown in (B).

We initially investigated whether the C-terminal region of IKKε is required for the induction of IFNβ promoter activity. To this end, we generated a series of C-terminal truncated forms of IKKε and compared the ability to activate IFNβ promoter with the corresponding fragments of TBK1 (Fig. 1A). As shown in Fig. 1B, the respective deletion of 37 and 80 residues at the TBK1 C-terminal (i.e. 1-692 and 1-649) induced IFNβ promoter activity in the same manner as wt TBK1 (1-729). The further deletion of 99 residues (1-550) resulted in the loss of promoter activity, in the case of the kinase activity defective K38A mutant. These results are in good accord with the previous observation that TBK1 truncation 1-643 is still dimeric, which configuration is needed to activate TBK1 [17]. In contrast to TBK1, the C-terminal deletion mutants of IKKε failed to activate the IFNβ promoter, even with a deletion of only 31 amino acids (aa) (1-685), indicating that the C-terminal region of TBK1 and IKKε plays a different role in the activation of downstream signaling, and this region in IKKε is indispensable for the activation of the IFNβ promoter in contrast to TBK1. To confirm this observation, we tested whether C-terminal deletion of IKKε affects endogenous IFNβ gene expression and found that wt IKKε, but not 1-685 and 1-640 mutants, induces IFNβ mRNA (Fig. 1C). Given that deletion of the C-terminal region in TBK1 and IKKε yielded a different effect on downstream signaling, we continued the investigation with a detailed domain analysis of IKKε. A series of IKKε mutants lacking a range of approximately 10 to 100 residues at the C-terminal were generated and the relative induction of IFNβ promoter activity was measured (Fig. 2A). As shown in Fig. 2B, the expression of wt IKKε as well as IKKε/1-705 activated the IFNβ promoter, while the promoter activity elicited by 1-685 or further truncated mutants was comparable to that of an empty vector or 1-716/K38A kinase defective mutant (Fig. 2B, top panel). These results indicate that the region between 686 and 705 contains an essential domain required for the induction of IFNβ promoter activity. We tested whether these truncated forms of IKKε induce the phosphorylation of endogenous IRF3 at Ser 386 and Ser 396, which is a hallmark of IRF3 transcriptional activity. Unexpectedly, three mutants (1-685, 1-671, and 1-657) that failed to activate the IFNβ promoter nevertheless induced both Ser 386 and Ser396 phosphorylation, although the phosphorylation signals were weaker than that of wt IKKε or 1-705. In contrast, 1-640 and 1-620 failed to phosphorylate IRF3, similar to the K38A mutant. These results indicate that in addition to 686-705, the 641-657 region of IKKε contains a novel functional domain involved in IRF3 phosphorylation (Fig. 2A). We also tested whether corresponding these functional domains in TBK1 are required for phosphorylation of IRF3. As shown in Fig. 2C, TBK1/1-692 as well as TBK1/1-649 normally induced IFNβ promoter activity and phosphorylation of IRF3 at Ser 386 and Ser 396 (Fig. 2C, left panels). Thus, in contrast to IKKε, the C-terminal region of TBK1 does not contain functional domains required for both activation of IRF3 and IFNβ promoter activity and is dispensable for activation of downstream signaling.

### Putative helical region in IKKε contributes to the IFNβ promoter activity

**Figure 3:**
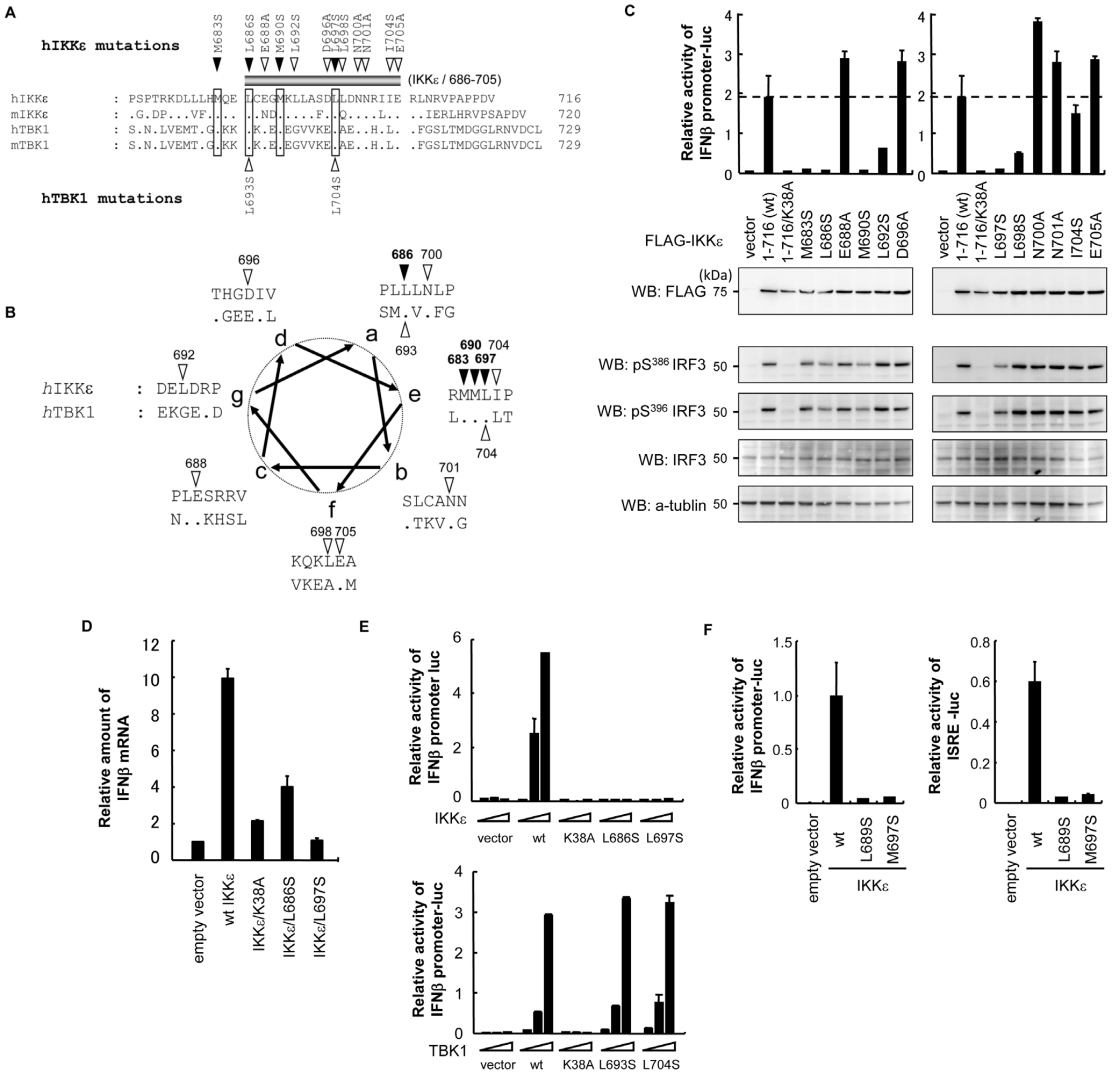
Mutation in the 686–705 region of IKKε, but not TBK1, had an effect on IFNβ promoter activity. (A) Alignment of the C-terminal regions of human and murine IKK-related kinases. The putative functional domain in IKKε that is required for IFNβ promoter activity (686–705) is shown. The amino acid substitutions introduced into IKKε (top) or TKB1 (bottom) are also shown. (B) A helical model of the C-terminal regions of IKKε and TBK1. The mutated residues are indicated with triangles. (C) 293T cells were transfected with the IFNβ promoter-luciferase reporter along with FLAG-tagged wt IKKε, kinase defective mutant or mutants with the indicated single amino acid substitutions. Cells were lysed 24 h post-transfection and luciferase activities were quantified by normalization with renilla luciferase activity. The values represent the average of three samples +/− SD. Cell lysates were also subjected to SDS-PAGE and Western blot with the indicated antibodies. (D) L cells were transfected with indicated plasmid and total RNA were prepared at 24 h post-transfection. Relative amount of IFNβ mRNA were quantified by using qRT-PCR by normalization with HPRT mRNA. The values represent the average of three samples +/− SD. (E) 293T cells were transfected with the IFNβ promoter-luciferase reporter along with an increasing amount of the wt or mutant forms of TKB1 and IKKε. Luciferase activities were measured as shown in (C). (F) 293T cells were transfected with the IFNβ promoter-luciferase or ISRE-luciferase reporter along with the wt or mutant forms of IKKε. Luciferase activities were measured as shown in (C).

The TBK1 and IKKε C-terminal regions are relatively conserved and certain residues are identical in the human and murine forms (Fig 3A). A TBK1 region corresponding to 686–705 of IKKε was previously proposed to form a helical structure, and L693 and K694 in TBK1 are involved in the induction of type I IFN promoter activity under physiological conditions [19]. A helical model of human IKKε and TBK1 prompted us to the observation that there are identical residues for the two kinases and that the hydrophobic residues are concentrated in a particular surface region (Fig. 3B, helix a and e). To investigate whether these residues are involved in IKKε function, IKKε and TBK1 mutants with a single aa substitution were generated (Fig. 3A and 3B, triangles), and the IFNβ promoter activity induced by these mutants was measured (Fig. 3C). Data showed that mutations in M683, L686, M690, and L697, all of which are located in a particular surface of the putative helical structure, failed to activate IFNβ promoter (Fig. 3C, the filled triangles in Fig. 3A and 3B). We also confirmed that L686S and L697S mutants of IKKε failed to induce endogenous IFNβ gene expression, like as kinase deficient K38A mutant (Fig. 3D). On the other hand, mutations in L693 and L704 of TBK1, corresponding to L686 and L697 of IKKε (Fig. 3A), had little, if any, effect on IFNβ promoter activity (Fig. 3E). These results are consistent with the previous observation that mutation in L693 or L704 of TBK1 did not affect the induction of type I IFN promoter activity in the overexpression system [19]. We also investigated whether these IKKε mutants phosphorylate IRF3 at Ser 386 and Ser 396 (Fig. 3C, the lower panels), and found that phosphorylation of endogenous IRF3 was observed not only in wt IKKε and the mutants that induced the IFNβ promoter, but also the mutants that failed to activate the IFNβ promoter, except for kinase defective mutant K38A. These results indicate that phenotypes of these mutants with single aa substitution are similar to that of C-terminal deletion mutant. The putative helical region in the IKKε C-terminal is required for activation of the IFNβ promoter, at least in this overexpression system, in contrast to TBK1 in which the counterpart is dispensable in the same experiment (Fig 3D) [19], demonstrating the functional difference between the C-terminal regions of TBK1 and IKKε in the type I IFN production pathway.

**Figure 4:**
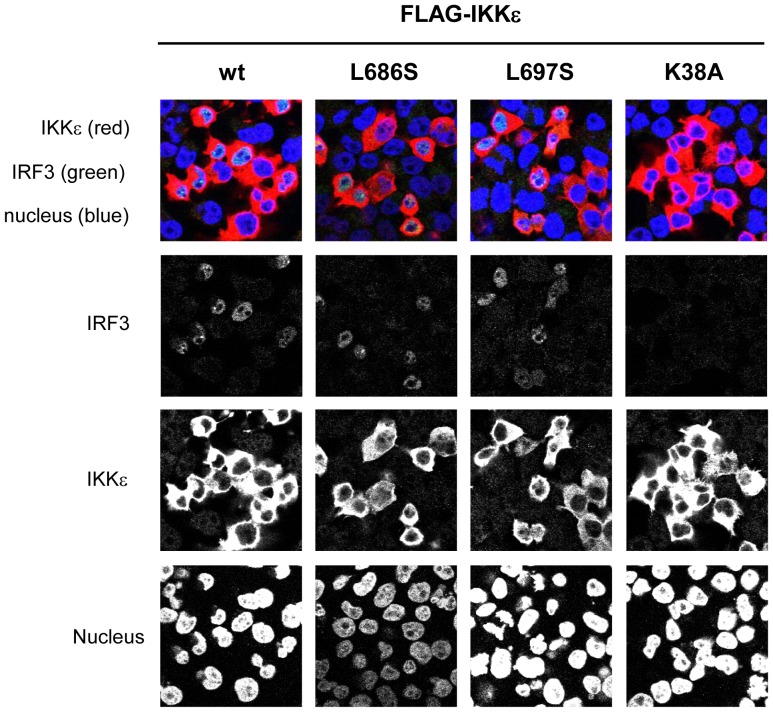
C-terminal mutants of IKKε induce nuclear translocation of IRF3. 293ET cells were transfected with FLAG-tagged wt IKKε or mutants as indicated on top and were fixed at 20 h post-transfection. Fixed cells were stained with anti-FLAG and anti-IRF3 antibodies and were observed by confocal microscopy. Green, red and blue fluorescence in merged figures (top panels) indicate the endogenous IRF3, FLAG-tagged IKKε and nucleus, respectively. Single channel images are also shown in lower panels.

At least three promoter elements, including interferon-sensitive response element (ISRE), κB, and activator protein 1 (AP-1), which is the binding site of IRFs, NFκB, and AP-1 transcription factors, respectively, were identified in IFNβ gene and these transcription factors orchestrate IFNβ gene expression [21], [22], [23]. To know lack of IFNβ activity in mutant IKKε was caused by abnormal activation of IRF3, we investigated whether IRF specific ISRE-reporter is activated by mutant IKKε. Again, IFNβ promoter was activated by wt IKKε, but not by L689S and M697S mutants (Fig 4F, left panel). In the same experimental condition, wt IKKε activated ISRE promoter (Fig 4F, right panel). Similar to IFNβ promoter, L689S and M697S mutants failed to activate ISRE. These results indicate that C-terminal mutants of IKKε could not activate IRF3 dependent transcription normally and that is probably the reason why these mutants failed to activate IFNβ promoter, although transcriptional activities of NFκB and AP-1 are also abnormal.

Given that C-terminal mutants which failed to induce IFNβ promoter activity, still have an ability to phosphorylate IRF3, we next investigated whether IRF3 activated by C-terminal mutants forms of IKKε were normally translocated to nucleus. Wt and mutant IKKε were transfected to 293ET cells and endogenous IRF3 in nucleus was observed by using confocal microscopy. As shown in Fig. 4, nuclear accumulation of IRF3 in L683S and L697S mutant expressing cells was observed, although cells in which IRF3 is accumulated in nucleus were extensively detected in wt IKKε expressing cells than that of these mutants. In this experimental condition, accumulation of IRF3 in nucleus was not observed in K38A mutant expressing cells at all. These results suggest that C-terminal region of IKKε is not essentially required for, at least, phosphorylation and nuclear translocation of IRF3 and therefore, lack of ability to induce IFNβ promoter activity may be caused by the deficiency of nuclear events following IRF3 nuclear translocation step.

### C-terminal region of IKKε is involved in the solid dimer formation

**Figure 5:**
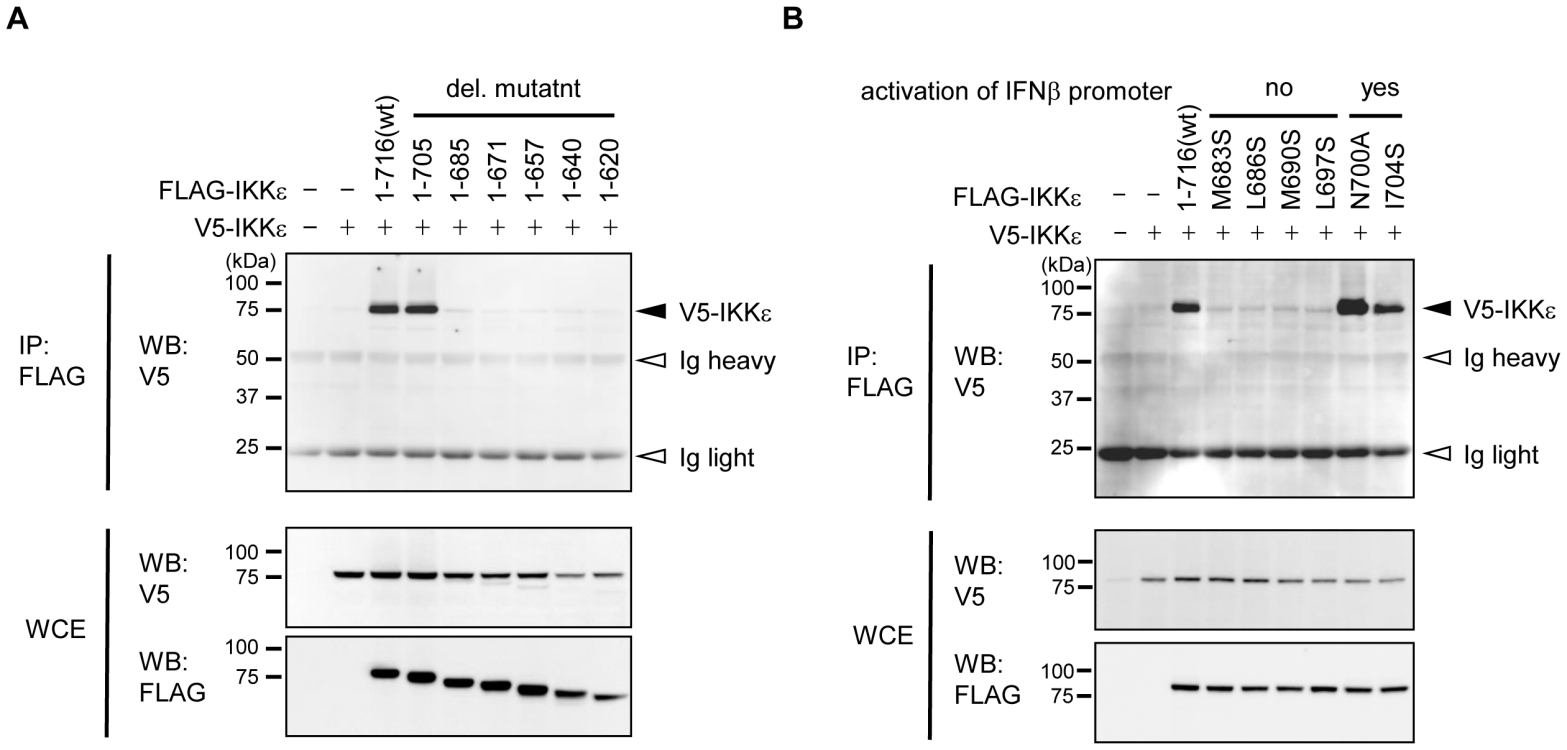
The C-terminal region of IKKε is involved in dimer formation. (A) V5-tagged IKKε and FLAG-tagged wt or the indicated C-terminal truncated mutants of IKKε were co-transfected into 293T cells. Cells were lysed 24 h post-transfection. Lysates were immunoprecipitated with FLAG-agarose and V5-tagged IKKε were detected with an anti-V5 antibody (top panel). V5 and FLAG-tagged IKKε in whole cell lysate (WCE) were also detected as indicated on the left. (B) V5-tagged IKKε and FLAG-tagged wt or IKKε mutants with a single amino acid substitution, as indicated at the top, were co-transfected into 293T cells. Immunoprecipitation was performed as in (A).

Mutations of TKB1 at the contact residues of dimerization led to a decrease of the auto-phosphorylation that enhances kinase activity, and thereby to a failure of the Ser 396 phosphorylation of IRF3 and ultimately of IFNβ gene expression [18]. We subsequently investigated whether a mutation in the C-terminal region of IKKε would have an effect on its dimer formation. V5-tagged wt and FLAG-tagged IKKε mutants were co-transfected into cells and dimer formation was assessed by immunoprecipitation (Fig. 5). As shown in Fig.5A, full length IKKε (1-716) was co-precipitated with full length or 1-705, while the association was greatly reduced in 1-685 or further deletion mutants. In the case of the substituted mutants, both the wt IKKε and the mutants inducing IFNβ promoter activity were strongly associated with wt IKKε, while the four mutants that failed to activate the IFNβ promoter were at almost undetectable levels (Fig 5B). These results suggest that the 686–705 region of IKKε includes the contact residues for dimer formation.

## Discussion

We have shown here that the C-terminal region of IKKε is involved in the induction of IFNβ promoter activity, while the corresponding region of TBK1 is not required. Mutant IKKε with a single aa substitution at C-terminal resulted in greatly decreased dimer formation and concomitantly lost the ability to induce IFNβ promoter activity, indicating dimerization of IKKε via C-terminal region is required for type I IFN production. In agreement with our observations of IKKε, mutations preventing dimerization of TBK1 resulted in a failure of type I IFN production [17], [18]. However, TBK1 forms a dimer without the C-terminal region, since the contact residues directly involved in TBK1 dimer formation are not located in the C-terminal, but rather, widely expanded to the KD, ULD and SSD [17], [18]. Like as IKKε, the critical dimer contact of IKKβis located in the C-terminal, and mutation of the critical C-terminal contact residues, these residues are not conserved in TBK1 and IKKε, resulted in a failure of dimerization of IKKβ and the loss of ability to phosphorylate its substrate [16]. Thus, even though IKKε is more closely related to TBK1 in terms of their aa sequences or substrates, the dimer organization of IKKε is apparently different from TBK1, and closer to that of IKKβ. It will be of interest to study the IKKε structure to elucidate the dimer organization and to compare it with TBK1 and IKKβ, especially with regard to the C-terminal.

Phospho-IRF3 signals were detected in cells transfected with various IKKε mutants lacking the ability to activate the IFNβ promoter. These results are quite unexpected, since it is well established that phosphorylated IRF3 is transcriptionally active and that any stimulation which induces the phosphorylation of IRF3 at Ser 386 and Ser 396 leads to an increase in IFNβ promoter activity [10]. Notably, the phospho-IRF3 signals in mutant IKKε expressing cells without any increased IFNβ promoter activity were clearly weaker than in the IFNβ promoter activated cells. Therefore, it is possible that the kinase activity of these mutants is insufficient to increase IFNβ promoter activity, although the kinase activity of these mutants is increased compared to the K38A mutant. Consistent with this idea, nuclear translocation of IRF3in cells expressing C-terminal mutated forms of IKKε was decreased compared to wt IKKε expressing cells (Fig. 4). Another possibility in loss of type I IFNβ promoter activity of IKKε C-terminal mutants is that certain Ser or Thr residues in IRF3, in addition to those in the C-terminal Ser/Thr rich region, including Ser 386 and Ser 396, may have to be phosphorylated for transcriptional activation, and these residues are not phosphorylated by these IKKε mutants [24]. Alternatively, some nuclear factors other than IRF3, which are also required for activation of IFNβ promoter activity, may not be activated by mutant IKKε and dimerization of IKKε is critically required for phosphorylation of the nuclear factors. In any, dimerization deficient IKKε still phosphorylates IRF3, indicating that dimerization is not essential in IKKε kinase activity for IRF3.
